# KHDRBS3 accelerates glycolysis and promotes malignancy of hepatocellular carcinoma via upregulating 14-3-3ζ

**DOI:** 10.1186/s12935-023-03085-4

**Published:** 2023-10-17

**Authors:** Mingda Zhao, Yibing Zhang, Longfei Li, Xiaobin Liu, Wenping Zhou, Chunhui Wang, Yufu Tang

**Affiliations:** 1 Department of Hepatobiliary Surgery, General Hospital of Northern Theater Command, 83#, Wenhua Road, Shenyang, Liaoning China; 2 Dalian Medical University, Dalian, Liaoning China; 3 Department of Medical Affairs, General Hospital of Northern Theater Command, Shenyang, Liaoning China

**Keywords:** Hepatocellular carcinoma, KHDRBS3, 14-3-3ζ, Glycolysis, Doxorubicin resistance

## Abstract

**Background:**

Primary hepatocellular carcinoma (HCC) is a malignancy with high morbidity and mortality. KH domain-containing, RNA-binding signal transduction-associated protein 3 (KHDRBS3) is an RNA-binding protein that is aberrantly expressed in multiple tumors; however, its expression and biological function in HCC have not been reported.

**Methods:**

KHDRBS3 knockdown and overexpression were performed using the lentiviral vector system to investigate the effects of KHDRBS3 on cell proliferation, apoptosis, chemoresistance, and glycolysis. Murine xenograft tumor models were constructed to study the role of KHDRBS3 on tumor growth in vivo. Furthermore, RNA-Pull Down and RNA immunoprecipitation were utilized to explore the interaction between KHDRBS3 and 14-3-3ζ, a phosphopeptide-binding molecule encoded by YWHAZ.

**Results:**

KHDRBS3 was highly expressed in human HCC tissues and predicted the poor prognosis of patients with HCC. Knockdown of KHDRBS3 exhibited a carcinostatic effect in HCC and impeded proliferation and tumor growth, reduced glycolysis, enhanced cell sensitivity to doxorubicin, and induced apoptosis. On the contrary, forced expression of KHDRBS3 expedited the malignant biological behaviors of HCC cells. The expression of KHDRBS3 was positively correlated with the expression of 14-3-3ζ. RNA immunoprecipitation and RNA pull-down assays demonstrated that KHDRBS3 bound to YWHAZ. We further confirmed that 14-3-3ζ silencing significantly reversed the promotion of proliferation and glycolysis and the inhibition of apoptosis caused by KHDRBS3 overexpression.

**Conclusions:**

Our findings suggest that KHDRBS3 promotes glycolysis and malignant progression of HCC through upregulating 14-3-3ζ expression, providing a possible target for HCC therapy.

**Supplementary Information:**

The online version contains supplementary material available at 10.1186/s12935-023-03085-4.

## Introduction

Liver cancer is a global problem that seriously threatens people’s lives and health and is expected to affect more than 1 million people every year by 2025 [1]. Primary hepatocellular carcinoma (HCC) is the most common pathological type of liver cancer (about 90%) [2]. HCC is the third leading cause of cancer death, with China accounting for more than half of all HCC cases worldwide [3]. Liver resection and transplantation are the most effective treatments for patients with early-stage HCC [4]. Since the disease is mostly asymptomatic in the early stages, most patients are diagnosed late and miss out on the best treatment time [5]. The Prognosis for advanced HCC remains poor; the five-year survival rate is 5% [6]. Although the survival rate of HCC has improved due to increased awareness and technological advances, the prognosis for HCC patients is still unsatisfactory. Therefore, novel potential therapeutic targets need to be identified urgently to expand treatment options and provide further insights into the pathogenesis of HCC.

14-3-3 is an acidic protein family ubiquitously expressed in mammals and other species, consisting of seven highly conserved isoforms (β, ε, γ, η, σ, τ, ζ) [7]. 14-3-3ζ proteins are able to bind a variety of signaling proteins with different functions, including kinases, phosphatases, and transmembrane receptors, and have pivotal roles in the regulation of cell signal transduction, cell proliferation and apoptosis [8]. 14-3-3ζ has been reported to promote the progression of multiple tumors, including HCC [9, 10]. For instance, 14-3-3ζ promoted cancer cell proliferation and enhanced resistance to sorafenib in HCC [11, 12]. Downregulation of 14-3-3ζ induced apoptosis in glioblastoma [13]. In addition, studies have shown that 14-3-3ζ increases glycolysis in pancreatic carcinoma cells [14]. However, its potential role in glycolysis of HCC and molecular regulatory mechanisms are still poorly understood.

KH domain-containing, RNA-binding signal transduction-associated protein 3 (KHDRBS3) is an RNA-binding protein that regulates alternative splicing of mRNAs to affect tumorigenesis [15]. In recent years, growing scientific researches have focused on the importance of KHDRBS3 in cancer. Zhang et al. found that specific knockout of KHDRBS3 regulated the expression of multiple downstream genes and inhibited the malignant progression of pancreatic cancer [16]. In colorectal cancer, abnormally high expression of KHDRBS3 is closely associated with multi-drug resistance [17]. Moreover, KHDRBS3 has been reported to promote glycolytic metabolism and chemoresistance to paclitaxel in ovarian cancer cells [18]. Nevertheless, the biological function, clinical significance and potential molecular mechanisms of KHDRBS3 in HCC remain poorly understood.

In the present study, we hypothesized that KHDRBS3 plays a role in HCC cell proliferation and glycolysis by regulating 14-3-3ζ expression. Our results showed that knockdown of KHDRBS3 suppressed proliferation, chemoresistance and glycolysis, and induced apoptosis of HCC cells, whereas overexpression of KHDRBS3 promoted malignant behaviors in HCC cells. We also found that KHDRBS3 bound to YWHAZ (encoding 14-3-3ζ) and upregulated 14-3-3ζ expression. The function of KHDRBS3/14-3-3ζ axis in proliferation and glycolysis may provide new therapeutic targets for HCC treatment.

## Methods

### Differential expression analysis

Differentially expressed genes (DEGs) were analyzed based on R software under the restrictive condition of p-value < 0.05 and |log2 fold change (FC)| > 1. Functional annotations of these DEGs were performed by Gene Ontology (GO) enrichment analysis with DAVID online tool (https://david.ncifcrf.gov/tools.jsp). GO was used to describe the properties of genes in three terms: cellular component (CC), biological process (BP), and molecular function (MF).

### Human samples

HCC tissues (n = 20) and para-tumor tissues (n = 20) were obtained from the General Hospital of Northern Theater Command and approved by the Ethics Committee of General Hospital of Northern Theater Command. This study was conducted in accordance with the guidelines of the Declaration of the World Medical Association of Helsinki. Written informed consent was obtained from the patients.

### Cell lines

Human HCC cell lines, Huh7 and SNU387, were obtained from iCell Bioscience Inc (iCell-h080, Shanghai, China) and Guangzhou saiku Biotechnology Co., Ltd (CC0112, Guangzhou, China), respectively. Huh7 cells were cultured in DMEM medium (iCell Bioscience Inc, iCell-128-0001, Shanghai, China) supplemented with 10% FBS (Tianhang, Huzhou, China). Cells were maintained in RPMI-1640 medium (Solarbio, Beijing, China) containing 10% FBS. The cells were placed in an incubator at 37 ℃ with 5% CO_2_.

### Cell treatment

Lentiviral knockdown and overexpression construct of KHDRBS3 were synthesized and inserted (General Biol, Chuzhou, China) into the pLVX-shRNA1 and pLVX-IRES-puro lentiviral vectors (YouBio, Changsha, China), respectively. The target sequences of human KHDRBS3 gene interference are as follows: KHDRBS3-shRNA-1, 5′-GCUGGGACAGAAAGUGUUAAU-3′; KHDRBS3-shRNA-2, 5′-GAAGCGUUUACAAGAAGAAAC-3′. Lentiviral infection was performed according to the manufacturer’s instructions. Cells with stable lentiviral expression were selected with puromycin (MACKLIN, Shanghai, China), and knockdown or overexpression efficiency of KHDRBS3 was assessed by Western blotting. To assess chemosensitivity, cells were treated with doxorubicin (MACKLIN, Shanghai, China) for 48 h after infection and then assayed for cell viability and apoptosis. For 14-3-3ζ knockdown, the siRNA targeting the 14-3-3ζ (14-3-3ζ^−si^) or its negative control (NC^− si^) was constructed and transfected into cells with Lipofectamine 3000 (Thermo Scientific, Pittsburgh, PA, USA) following the instructions provided by the manufacturer.

### Subcutaneous xenograft tumor model

Nude mice (7–8 weeks old) were housed in a standard laboratory environment with free access to water and food. Huh7 cells (5 × 10^6^) were resuspended and injected subcutaneously into the right leg femoral root of nude mice. Tumor volumes were monitored every three days and calculated according to the formula: volume = 1/2 (width^2^ × length). Tumors were harvested at the end of the experiment, photographed, and fixed in formaldehyde or stored at -70 °C for subsequent experiments.

### MTT assay

The suspension containing 5000 cells was added to each well of 96-well plates. For detection, the medium in each well was replaced with 50 µL MTT solution (KeyGen Biotech, Nanjing, China). After 4 h, the MTT solution was removed and 150 µL of DMSO (KeyGen Biotech, Nanjing, China) was added to dissolve the crystals. The absorbance was measured at 490 nm.

### Annexin V-PI staining of apoptotic cells

Stably infected Huh7 and SNU387 cells were seeded into 6-well plates at a density of 5 × 10^5^. In certain experiments, cells were then treated with 1 µmol/L doxorubicin for 48 h. Cell were harvested, washed with PBS, and resuspended in 500 µL Binding Buffer. The percentage of apoptotic HCC cells was measured by flow cytometry (Agilent Technologies, NovoCyte, Santa Clara, CA, USA) using the Annexin-V-FITC/PI Apoptosis Kit (KeyGen Biotech, Nanjing, China). The apoptosis rate was the sum of the early apoptosis rate and the late apoptosis rate.

### Measurements of glucose consumption and lactate production

Cell supernatants were collected and glucose levels and lactate production were detected using commercially available kits (Jiancheng Bioengineering Institute, Nanjing, China). Glucose consumption was the difference between the glucose concentration in the unseeded cell culture medium and the cell culture medium.

### Immunohistochemical (IHC) analysis

Fixed HCC tissues were embedded in paraffin and then cut into 5 μm slices. The slices were deparaffinized and rehydrated. Antigen retrieval was performed with citrate buffer for 10 min, followed by blocking endogenous peroxidase activity for 15 min in 3% H_2_O_2_ (Sinopharm, Shanghai, China). Slides were blocked in 1% BSA (Sangon, Shanghai, China) for 15 min and then incubated with KHDRBS3 antibody (Santa Cruz Biotechnology, sc-374,461, Shanghai, China) diluted (1:50) in PBS at 4 °C overnight. Slices were incubated with Goat anti-Mouse IgG Secondary Antibody, HRP conjugate (Thermo Scientific, 31,430, Pittsburgh, PA, USA) at a dilution of 1:500 for 1 h, followed by counterstaining with hematoxylin (Solarbio, Beijing, China). The Olympus DP73 microscope camera system (Tokyo, Japan) was used to capture IHC images.

### Colony formation assay

Four hundred cells were seeded in petri dishes and cultured for two weeks. Clones were washed twice with PBS and then stained with Wright-Giemsa Stain (Jiancheng Bioengineering Institute, Nanjing, China). The excess dye was carefully rinsed off with water and then the cells were counted.

### Quantitative real-time-PCR (qPCR)

Total RNA was extracted from HCC tissues and cells using TRIpure (Bioteke, Beijing, China) and reverse transcribed to cDNA. qPCR was run on a Exicycler 96 (Bioneer, Daejeon, Korea) using SYBR Green (Solarbio, Beijing, China). Data were calculated by the 2^−ΔΔCT^ method and normalized to the expression of GAPDH. The primers used are listed in Table 1.

**Table 1:** Sequence of primers for qRT-PCR

Gene	Forward 5’ − 3’	Reverse 5’ − 3’
c-Myc	CACCCTTCTCCCTTCGG	CAGTCCTGGATGATGATGTTT
CRABP1	TCGGAGAAGGCTTTGAGG	CACGGGTCCAGTAGGTTTT
DLL4	TGGGTCAGAACTGGTTATTG	GCCCGAAAGACAGATAGG
GLUT1	TGTGCTCCTGGTTCTGTTCT	GCTCCTCGGGTGTCTTGT
HOXB9	CGTCCGTCTACCACCCTTAC	TTGTCCTCGCTTCCTTCG
LDHA	ATTTGGTCCAGCGTAAC	CCACTCCATACAGGCAC
YWHAZ	TGAGACGGAGCTAAGAG	CCAAGTAACGGTAGTAATC
KHDRBS3	AAAGTGGAGAAGCGAAGT	ATAATCAGGGATGAGGAAC
GAPDH	GACCTGACCTGCCGTCTAG	AGGAGTGGGTGTCGCTGT

### Western blot analysis

Total proteins were extracted using RIPA buffer (Solarbio, Beijing, China) containing 1% PMSF (Solarbio, Beijing, China). Protein concentrations were determined using the BCA protein concentration assay kit (Solarbio, Beijing, China). Protein samples (10–20 µg) were diluted to the same volume with Loading Buffer and then separated by 5% SDS-PAGE before transferring to PVDF membranes (Millipore, Billerica, MA, US). The membranes were blocked in 5% skim milk (Sangon, Shanghai, China) for 1 h and then incubated with the specific primary antibodies overnight at 4 °C. The primary antibodies are as follows: KHDRBS3 (Santa Cruz Biotechnology, sc-374,461, Shanghai, China; 1:500); c-Myc (ABclonal, A1309, Shanghai, China; 1:500); GLUT1 (Affbiotech, AF5462, Changzhou, China; 1:500); LDHA (ABclonal, A1146, Shanghai, China; 1:1000); 14-3-3ζ (Affbiotech, AF6356, Changzhou, China; 1:1000); GAPDH (Proteintech, 60004-1-Ig, Wuhan China; 1:10000). After incubation with HRP-conjugated secondary antibodies (Solarbio, Beijing, China), the bands were visualized using ECL Plus kit (Solarbio, Beijing, China).

### RNA-Pull down assay

RNA pull-down assay was performed with Magnetic RNA-Protein Pull-Down Kit (Pierce Biotechnology, Rockford, IL, USA) according to the protocol of manufacturer. The enriched proteins were recovered and detected by Western blotting.

### RNA immunoprecipitation (RIP)

RIP assay was performed using EZ-Magna RIP Kit (Millipore, Billerica, MA, US) following the manufacturer’s instructions. Co-precipitated RNA was determined by Real-time-PCR.

### KHDRBS3 expression and prognostic value analysis

The expression of KHDRBS3 in liver cancer samples (n = 369) and normal samples (n = 160) was analyzed using the GEPIA database (http://gepia2.cancer-pku.cn/#index). The prognostic value of KHDRBS3 was evaluated in terms of overall survival (OS), recurrence-free survival (RFS), progression-free survival (PFS), and disease-specific survival (DSS) in patients with liver cancer using the Kaplan-Meier plotter (http://kmplot.com/analysis/index.php?p=background).

### Statistical analysis

Data were analyzed by GraphPad software 7.0 (GraphPad Prism, La Jolla, CA, USA) and expressed as mean ± standard deviation (SD). Unpaired Student’s t-test and ANOVA were used for comparisons between groups. P < 0.05 was considered statistically significant.

## Results

### Identification and functional annotation of DEGs in HCC

**Fig. 1:**
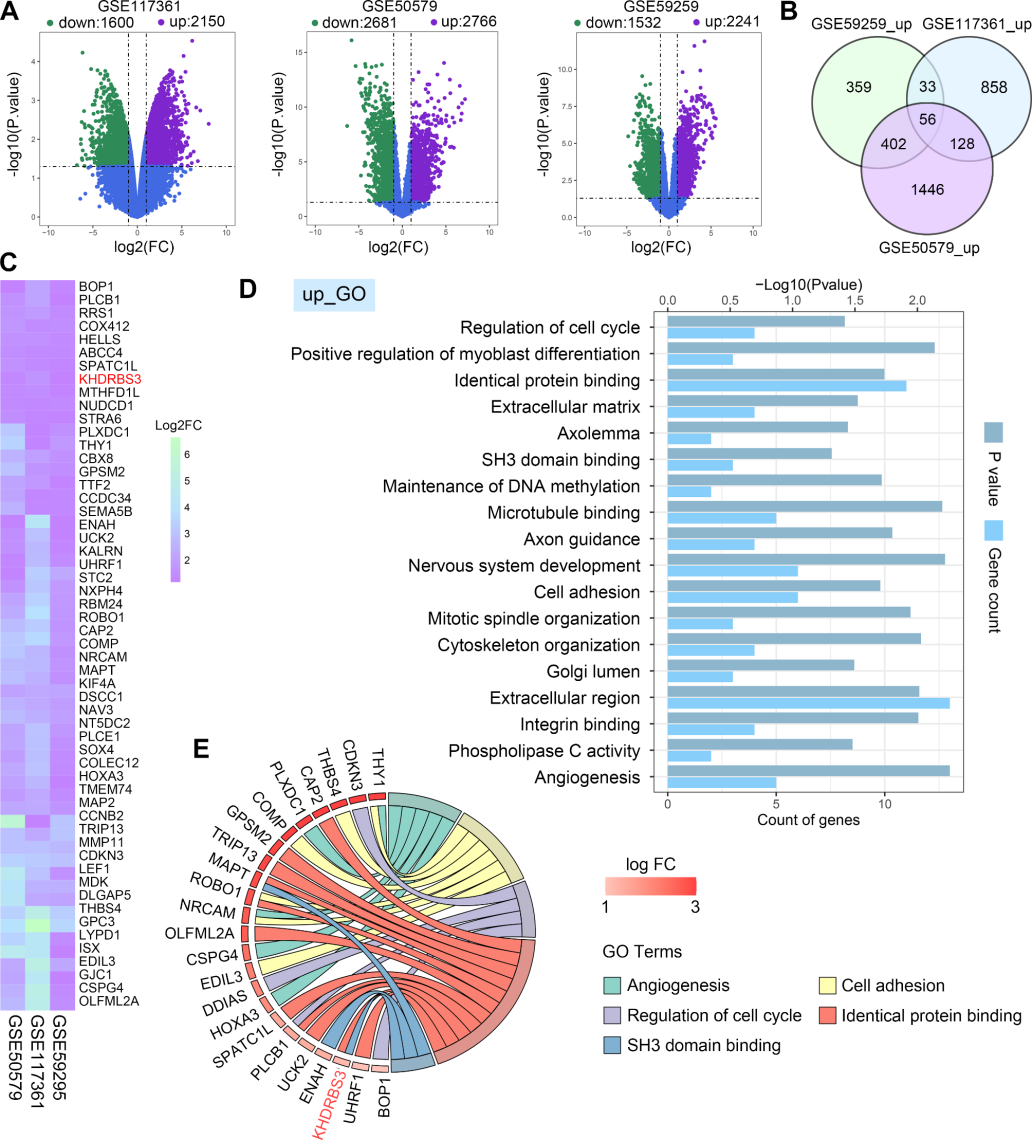
Identification and functional annotation of differentially expressed genes in HCC. (**A**) Volcano plots show the DEGs in GSE117361, GSE50579 and GSE59259 datasets. (**B**) The Venn diagram suggests the overlapping upregulated DEGs among three datasets. (**C**) The heatmap diagram shows the overlapping upregulated DEGs. (**D**) GO analysis for the overlapping upregulated DEGs. (**E**) The chord plot shows the GO function enrichment. The left half ring shows the upregulated DEGs and the right half ring shows GO terms

The DEGs from three GEO datasets were identified and visualized as volcano plots (Fig. 1A). The Veen diagram showed that there were 56 overlapping upregulated DEGs and 83 downregulated DEGs in these datasets (Fig. 1B and Fig. S1A). The overlapping upregulated or downregulated DEGs was displayed in heatmap (Fig. 1C and Fig. S1B). GO enrichment analysis were performed to characterize the possible biological functions of these upregulated (Fig. 1D-E) or downregulated DEGs (Fig. S1C-D). The upregulated DEGs were enriched in the “extracellular region”, “regulation of cell cycle”, “cell adhesion”, and “identical protein binding”, which include KHDRBS3. These results suggest that the upregulated DEGs may be associated with cancer progression.

### KHDRBS3 is elevated in human HCC tissues and associated with poor prognosis in HCC patients

**Fig. 2:**
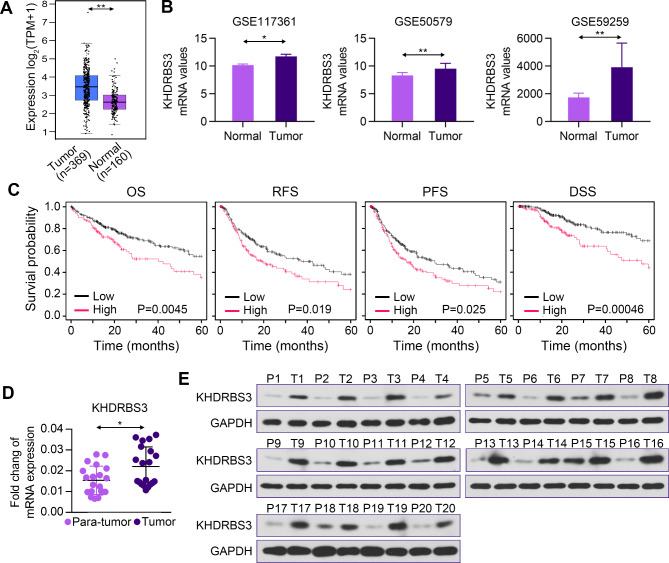
KHDRBS3 is elevated in human HCC tissues and is associated with poor clinal outcomes. (**A**) The expression profiles of KHDRBS3 gene in HCC on GEPIA database. (**B**) The expression of KHDRBS3 gene in three GEO datasets. (**C**) The overall survival (OS), recurrence-free survival (RFS), progression-free survival (PFS) and disease-specific survival (DSS) analysis in HCC patients with different KHDRBS3 expression. (**D-E**) KHDRBS3 expression in human HCC and adjacent tissues were detected by qPCR and Western blotting. Error bars represent standard deviation. ^*^P < 0.05 vs. Para-tumor group; ^**^P < 0.01 vs. Normal group

**Fig. 3:**
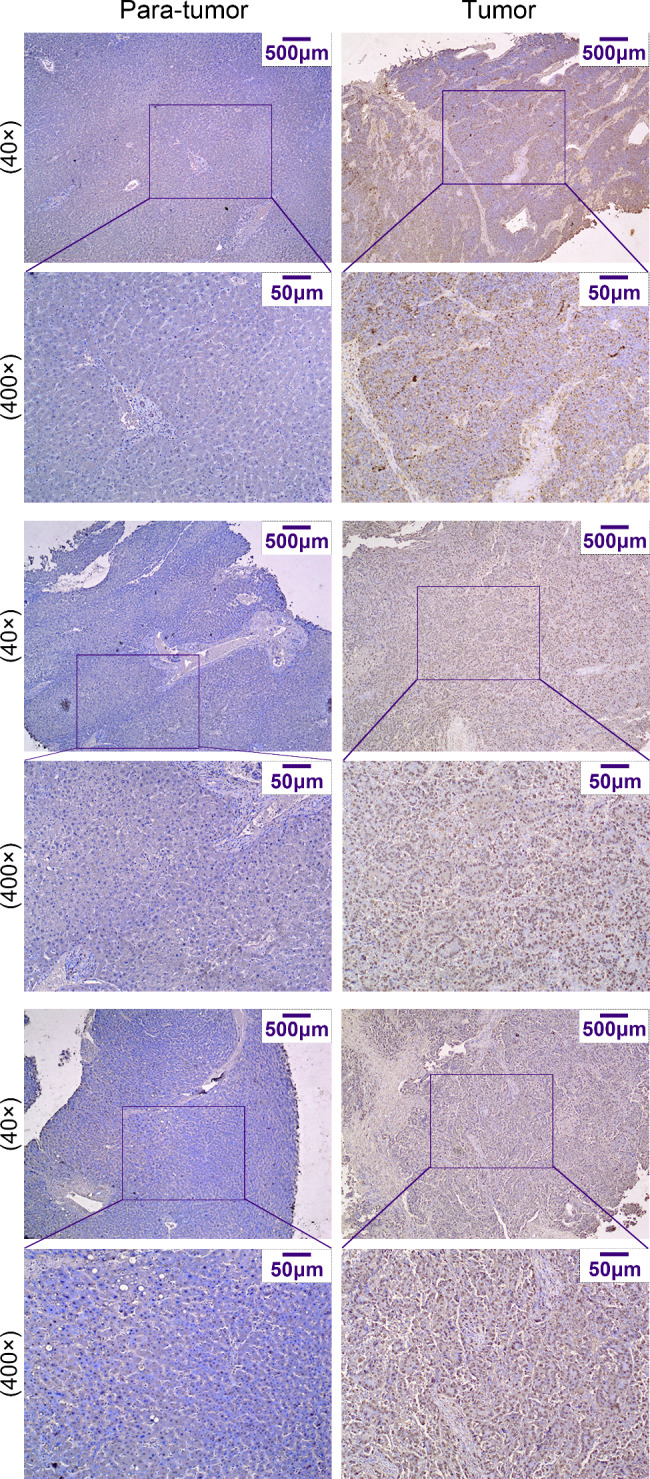
Representative images of KHDRBS3 immunohistochemical staining in HCC and adjacent tissues

Considering the results of bioinformatics analysis and previous researches, we decided to further study KHDRBS3. GEPIA database analysis indicated that the expression of KHDRBS3 in HCC tissues was significantly higher than that in normal tissues (Fig. 2A). Moreover, KHDRBS3 expression was higher in tumor tissues compared to normal tissues in the three GEO datasets (Fig. 2B). Patients with high expression of KHDRBS3 had worse prognosis than those with low expression of KHDRBS3 (Fig. 2C). To assess the clinical significance of KHDRBS3 expression in HCC, KHDRBS3 expression was examined in human HCC and precancerous tissues. The mRNA and protein levels of KHDRBS3 were increased in HCC tissues (Fig. 2D-E). IHC staining showed high expression of KHDRBS3 in HCC tumors (Fig. 3). These results suggest that KHDRBS3 expression is elevated in human HCC and is associated with poor prognosis.

**KHDRBS3 promotes proliferation of HCC cells** in vitro **and** in vivo.

**Fig. 4:**
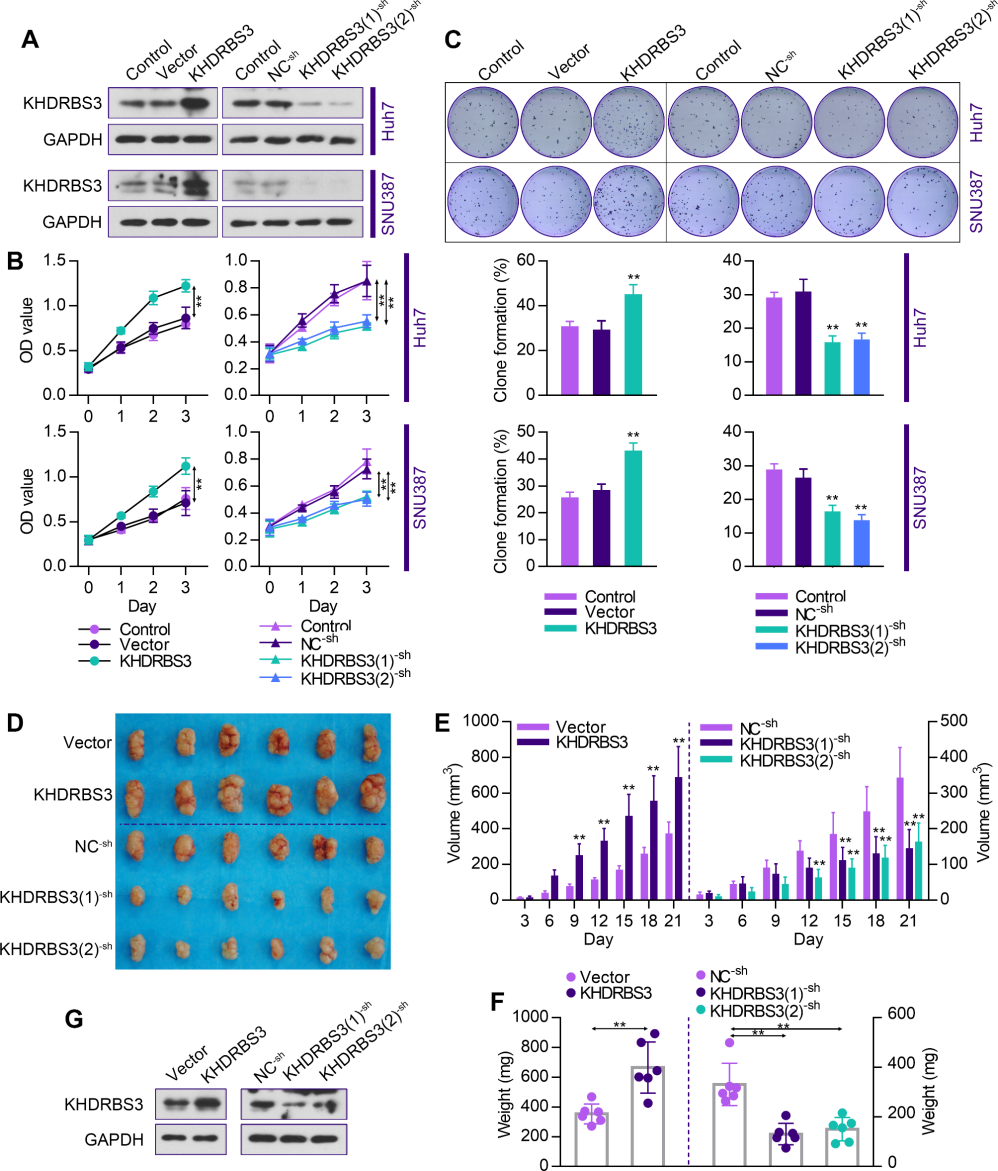
KHDRBS3 promotes cell proliferation in vitro and tumor growth in vivo. (**A**) The efficiency of knockdown and overexpression of KHDRBS3 was examined by Western blotting analysis. (**B-C**) MTT cell proliferation assay and colony formation assay in indicated cells. (**D-F**) Image, volume and weights of xenograft tumors derived from the Huh7 cells. (**G**) The protein levels of KHDRBS3 expression in xenograft tumors. Error bars represent standard deviation. ^**^P < 0.01 vs. Vector group or NC^− sh^ group

To determine the impact of KHDRBS3 in HCC cell proliferation, KHDRBS3-overexpression and KHDRBS3-knockdown cell lines were established in Huh7 and SUN387 cells and the efficiency was confirmed by Western blotting (Fig. 4A). As shown in Fig. 4B and C, overexpression expression of KHDRBS3 accelerated the proliferation and colony formation of both Huh7 and SUN387 cells in vitro. On the contrary, the proliferation and colony formation of these cells were suppressed upon KHDRBS3 silencing. To explore the role of KHDRBS3 in tumor growth in vivo, we established the subcutaneous xenograft tumor model and monitored the tumor growth. Overexpression of KHDRBS3 promoted tumor growth (Fig. 4D-F), while KHDRBS3 silencing inhibited tumor progression. Western blotting analysis revealed that KHDRBS3 expression was increased in KHDRBS3-overexpression tumors and decreased in KHDRBS3-knockdown tumors (Fig. 4G). Collectively, the above data indicate that KHDRBS3 promotes HCC cell proliferation in vitro and tumor growth in vivo.

### KHDRBS3 downregulation induces apoptosis and suppresses glycolysis of HCC cells

**Fig. 5:**
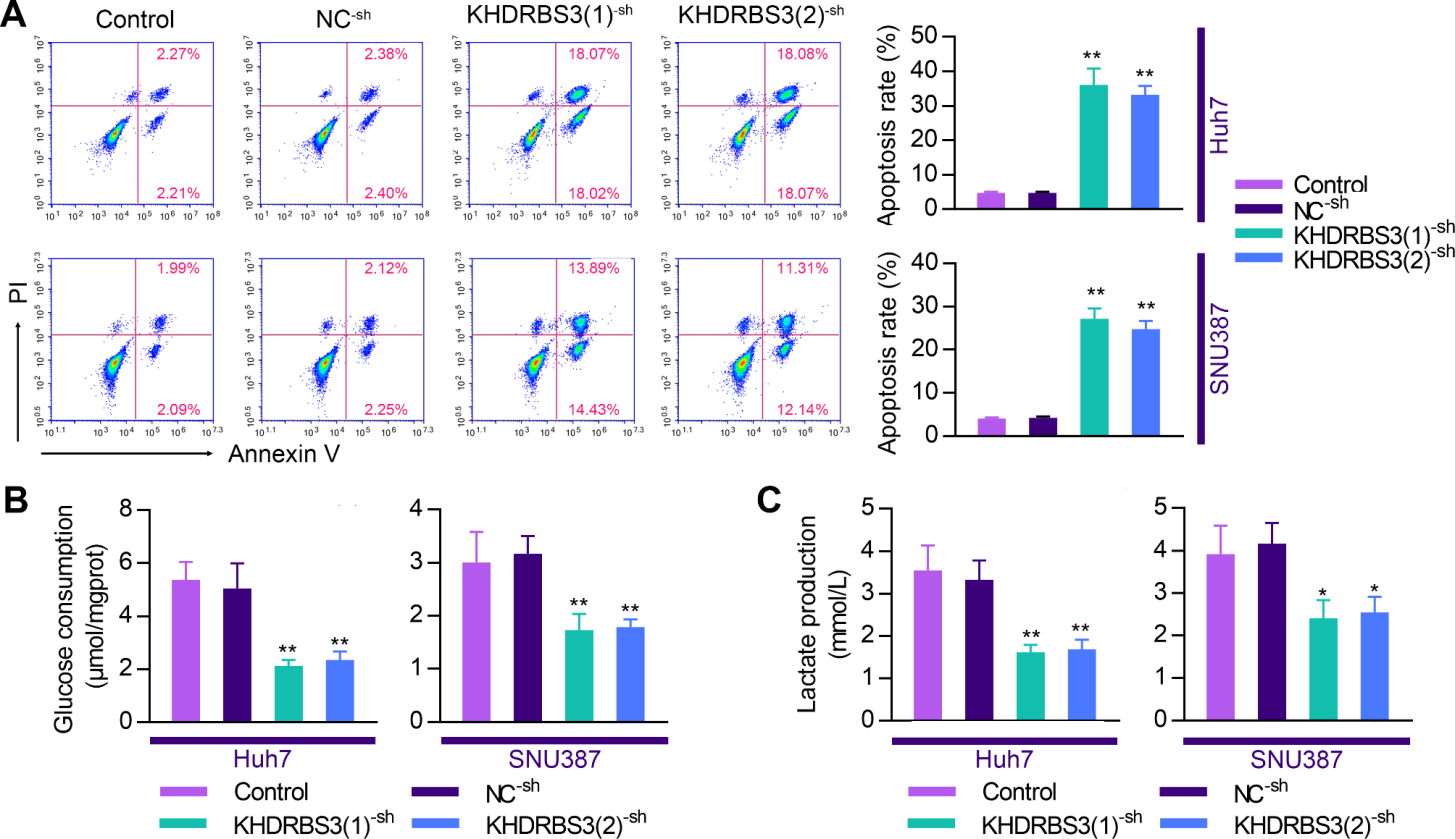
The knockdown of KHDRBS3 induces apoptosis and inhibits glycolysis of HCC cells. (**A**) Apoptosis was analyzed using flow cytometry with Annexin V-PI staining double staining. (**B-C**) Measurement of glucose consumption and lactate production in cell culture medium. Error bars represent standard deviation. ^*^P < 0.05, ^**^P < 0.01 vs. NC^− sh^ group

Subsequently, we elucidated the effects of KHDRBS3 silencing on the apoptosis and glycolysis of HCC cells. First, we observed that silencing of KHDRBS3 profoundly increased apoptosis of Huh7 and SUN387 cells (Fig. 5A). Furthermore, KHDRBS3 knockdown resulted in decreased glucose consumption and lactate production in both cells lines (Fig. 5B-C), indicating that KHDRBS3 silencing impedes glycolysis in HCC cells.

### KHDRBS3 enhances chemoresistance of HCC cells

**Fig. 6:**
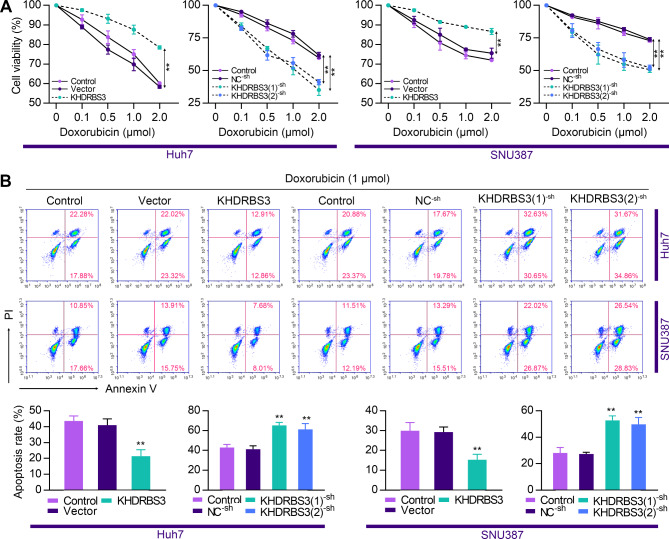
KHDRBS3 enhances chemoresistance of HCC cells. (**A**) Cell viability of HCC cells upon treatment with different concentrations of doxorubicin was assessed by MTT. (**B**) Flow cytometry analysis of indicated cells with doxorubicin (1 µmol) treatment. Error bars represent standard deviation. ^**^P < 0.01 vs. Vector group or NC^− sh^ group

We then evaluated the effect of KHDRBS3 on the sensitivity to doxorubicin in HCC cells. The results of MTT assay demonstrated that KHDRBS3 overexpression significantly increased the viability of HCC cells treated with various concentrations of doxorubicin (Fig. 6A). The opposite result was observed in KHDRBS3-silenced cells. In addition, KHDRBS3-overexpressing cells had a low rate of apoptosis (Fig. 6B), whereas the percentage of apoptotic cells increased after KHDRBS3 downregulation. Taken together, these results suggest that KHDRBS3 enhanced the resistance of HCC cells to doxorubicin.

### KHDRBS3 binds with YWHAZ and upregulates its expression

**Fig. 7:**
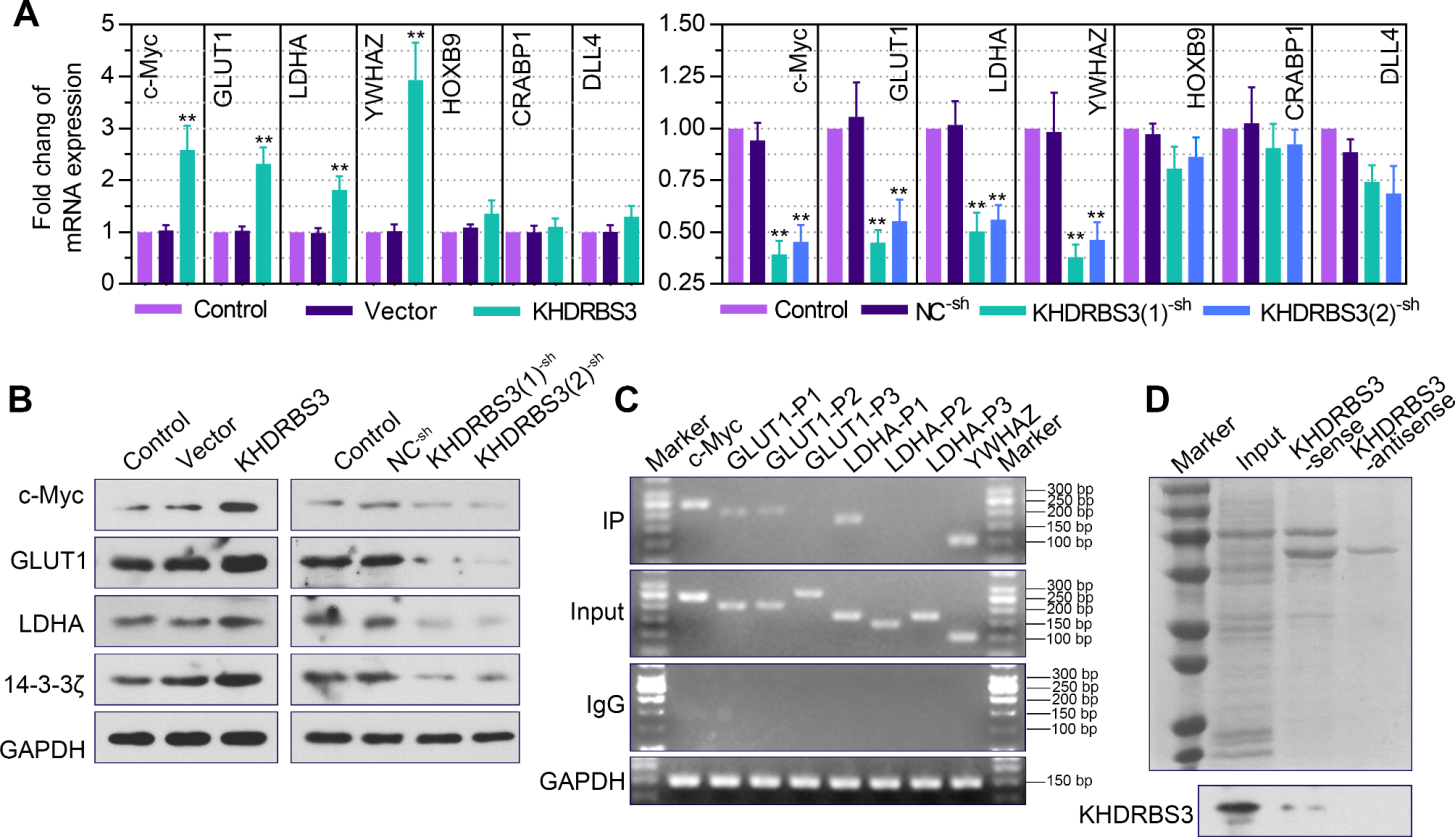
KHDRBS3 binds to YWHAZ and upregulates its expression. (**A**) Relative mRNA levels of c-Myc, GLUT1, LDHA, YWHAZ, HOXB9, CRABP1 and DLL4 in stable KHDRBS3 knockdown or overexpressing Huh7 cells. (**B**) Protein levels of c-Myc, GLUT1, LDHA and 14-3-3ζ in infected Huh7 cells were evaluated by western blotting. (**C**) RIP assay was performed to detect the enrichment of c-Myc, GLUT1, LDHA and YWHAZ in the anti-KHDRBS3 IP group. (**D**) RNA pull-down assay determined the binding between KHDRBS3 and YWHAZ. Error bars represent standard deviation. ^**^P < 0.01 vs. Vector group or NC^− sh^ group

To understand the mechanism by which KHDRBS3 regulates the malignant behavior of HCC cells, we investigated the changes in the expression of several downstream factors in Huh7 cells after KHDRBS3 overexpression or silencing. Among these factors, the mRNA levels of c-Myc, GLUT1, LDHA and YWHAZ were significantly elevated after KHDRBS3 upregulation and significantly decreased after KHDRBS3 downregulation (Fig. 7A), and their positive correlation with KHDRBS3 was further verified at the protein expression level (Fig. 7B). RIP results showed that c-Myc, GLUT1, LDHA and YWHAZ were enriched in the anti-KHDRBS3 IP group (Fig. 7C). The regulatory relationship of KHDRBS3 on c-Myc has been confirmed [17]. And it has been reported that decreased expression of 14-3-3ζ resulted in decreased expression of GLUT1, LDH and c-Myc [14]. We hypothesized that KHDRBS3 regulated GLUT1, LDH and c-Myc might be mediated by 14-3-3ζ, so we next focus on verifying the relationship between 14-3-3ζ and KHDRBS3. RNA pull-down analysis indicated that KHDRBS3 was bound to YWHAZ (Fig. 7D). These results confirm that KHDRBS3 binds to YWHAZ and promotes its expression.

### Inhibition of 14-3-3ζ reverses the effects of KHDRBS3 overexpression on malignant phenotypes of HCC cells

**Fig. 8:**
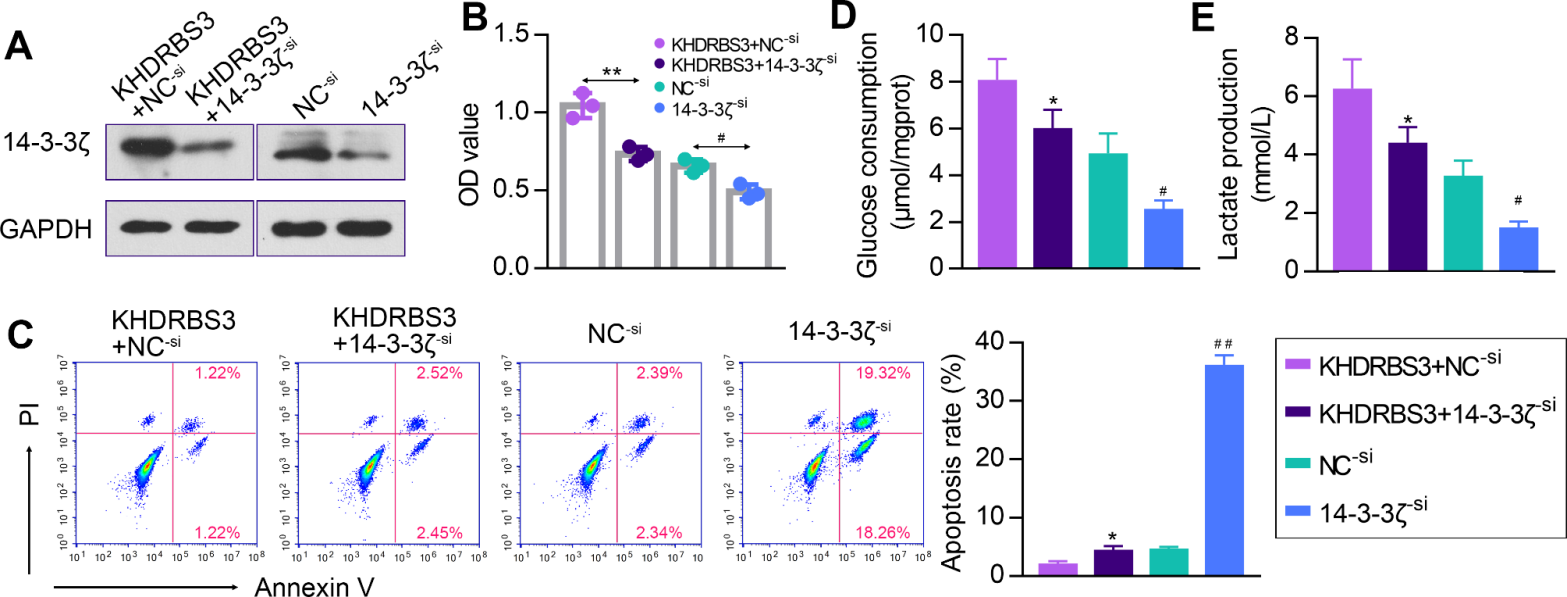
KHDRBS3 promotes malignant progression of HCC cells via regulating 14-3-3ζ expression. (**A**) 14-3-3ζ protein levels in Huh7 cells were measured by western blotting. (**B**) Cell proliferation was assessed using MTT assay. (**C**) Cell apoptosis was analyzed by flow cytometry. (**D-E**) Glucose consumption and lactate production were measured in Huh7 cells. Error bars represent standard deviation. ^*^P < 0.05, ^**^P < 0.01 vs. KHDRBS3 + NC^− si^ group; ^#^P < 0.05, ^##^P < 0.01 vs. NC^− si^ group

Furthermore, we performed rescue experiments to verify the impacts of KHDRBS3/14-3-3ζ axis on HCC cell proliferation, apoptosis and glycolysis. Western blotting revealed that the protein expression levels of 14-3-3ζ were upregulated in KHDRBS3 overexpressing cells and reduced after transfection with 14-3-3ζ siRNA (Fig. 8A). 14-3-3ζ silencing attenuated the promotion of Huh7 cell proliferation by KHDRBS3 overexpression (Fig. 8B). Apoptosis was significantly increased upon downregulation of 14-3-3ζ (Fig. 8C), and 14-3-3ζ silencing counteracted the inhibitory effect of KHDRBS3 overexpression on apoptosis. Moreover, the increased glucose consumption and lactate production were reduced in KHDRBS3 overexpressing Huh7 cells by knockdown of 14-3-3ζ (Fig. 8D-E). These findings demonstrate that KHDRBS3 facilitates malignant progression of HCC cells via upregulating the expression of 14-3-3ζ.

## Discussion

**Fig. 9:**
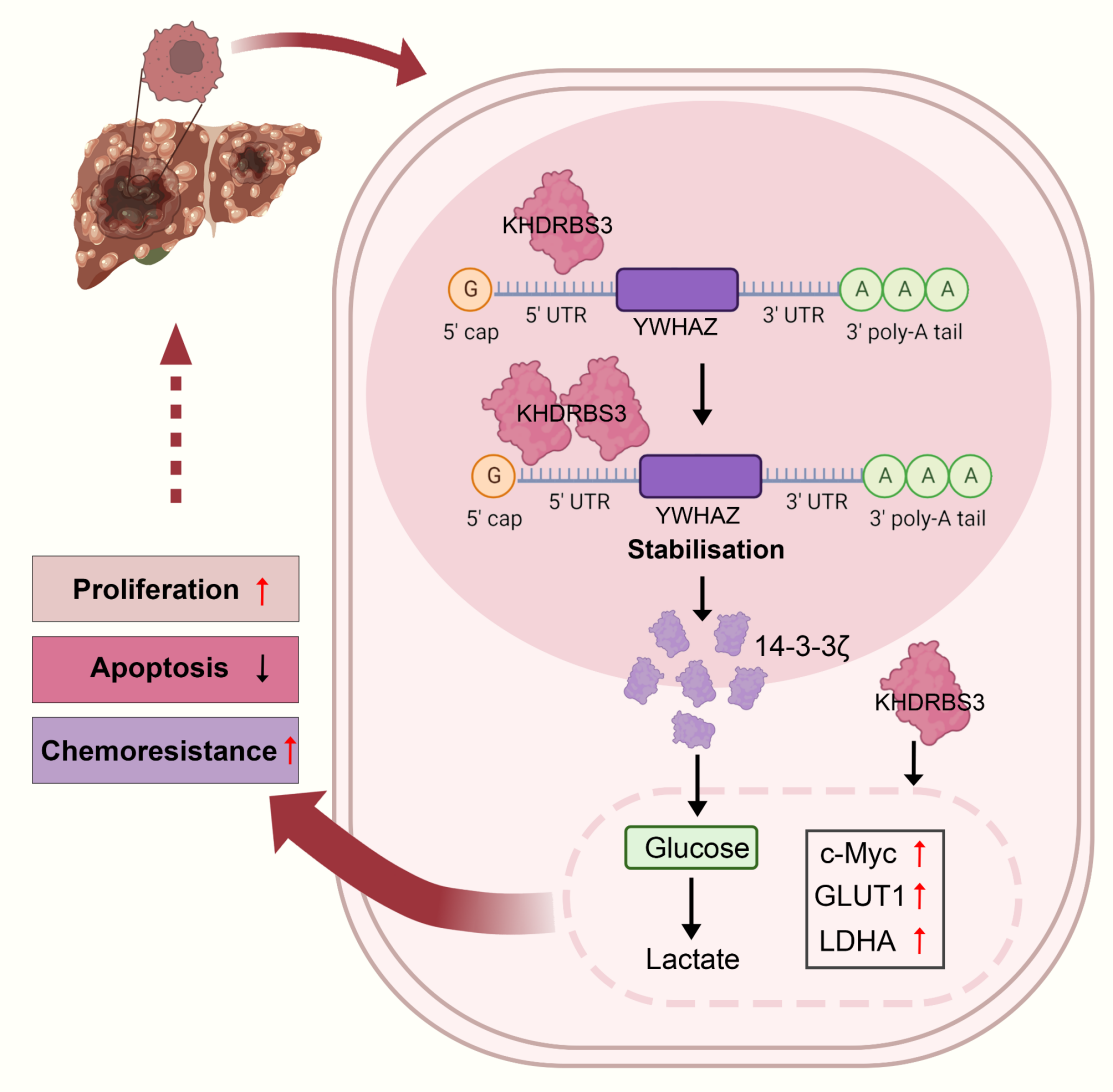
Diagram of the mechanism of KHDRBS3 promoting HCC progression. KHDRBS3 upregulates 14-3-3ζ expression by binding to YWHAZ, resulting in promotion of glycolysis, cell proliferation, chemoresistance and inhibition of apoptosis

HCC is a worldwide malignant tumor with high recurrence rate and poor prognosis. Chemotherapy resistance frequently occurs in HCC and needs to be improved [18]. Although many studies have indicated KHDRBS3 is involved in the progression of multiple malignant tumors [17, 18], few studies have been published on the function and molecular mechanisms of KHDRBS3 in HCC. In this study, KHDRBS3 was upregulated in HCC tumor tissues and predicted a poor prognosis. Overexpression of KHDRBS3 promoted proliferation of HCC cells in vitro and tumor growth in vivo and enhanced cell resistance to doxorubicin, whereas silencing of KHDRBS3 reversed these effects. Silencing of KHDRBS3 induced apoptosis and decreased glucose consumption and lactate production. The main mechanism is that KHDRBS3 binds to YWHAZ and regulates cell proliferation, apoptosis, and glycolysis in HCC cells by upregulating 14-3-3ζ expression (Fig. 9). Our study may provide a potential target for HCC treatment.

Aberrant expression of KHDRBS3 in different tumors has been reported [17, 19], but its expression profile in HCC has not been clarified. Thus, we examined the expression of KHDRBS3 in the tumors and adjacent noncancerous tissues of HCC patients, and the results demonstrated that the expression of KHDRBS3 was elevated. Consistent with clinical outcomes in other cancers [17, 18], high levels of KHDRBS3 predicted a poor prognosis for HCC patients. These results implicate the oncogenic potential of KHDRBS3 in HCC. Conversely, KHDRBS3 expression was associated with improved survival in breast cancer [19]. Furthermore, previous studies have shown that KHDRBS3 promotes cancer cell proliferation. Sernbo et al. revealed that KHDRBS3 promoted anchorage-independent proliferation by regulating CD44 expression and the Wnt signal pathway [17]. KHDRBS3 promoted xenograft tumor growth in vivo, and its downregulation induced apoptosis of ovarian cancer cells [18]. Consistent with these observations, our study revealed that KHDRBS3 overexpression promoted proliferation of HCC cells in vitro and in vivo, whereas downregulation of KHDRBS3 suppressed cell proliferation.

Increased glucose uptake and aerobic glycolysis are major events in glucose metabolism in cancer cells. Aerobic glycolysis is the process of metabolizing glucose to lactic acid [20]. The growing amount of evidence showed a correlation between glycolysis and chemotherapeutic drug resistance in cancer cells [21, 22]. In HCC cells, increased levels of aerobic glycolysis have been shown to correlate with drug resistance [23]. Inhibition of glycolysis may sensitize HCC cells to doxorubicin, a chemotherapeutic drug widely used to treat HCC [24]. A recent pivotal study revealed that KHDRBS3 plays an important role in regulating drug resistance in cancer cell [17]. KHDRBS3 promoted aerobic glycolysis and resistance to paclitaxel in cancer cells [18]. Rapidly proliferating cancer cells require more energy in addition to maintaining their own metabolic homeostasis. ATP and other intermediates produced by aerobic glycolysis may provide conditions for cancer cell proliferation. Glucose uptake provides sufficient feedstock for glycolysis to facilitate ATP production [25]. In our observation, KHDRBS3-silenced cells exhibited low lactate production and glucose uptake and attenuated resistance to doxorubicin, suggesting that knockdown of KHDRBS3 reduces the glycolysis level and attenuates drug resistance in HCC cells.

RNA-binding proteins play important roles in various cellular processes, including transcription, capping, mRNA splicing, polyadenylation, RNA stability, and modification [26]. KHDRBS3 shares the same basic structure as Sam68, with RNA binding activity and signaling properties [26]. KHDRBS3 participates in tumorigenesis and progression through its binding partners. For instance, KHDRBS3 was found to interact with FBXO32 mRNA to promote gastric cancer progression [27]. KHDRBS3 is upregulated by SALL4 as a splicing factor for CD44, which enhances stemness in breast cancer cells. [15]. In addition, c-Myc is an oncogene that regulates glycolysis-related genes such as GLUT1 and LDHA [14]. Consistent with our results, KHDRBS3 has been found to upregulate these glycolysis-related genes [14]. Besides, we further determined that KHDRBS3 bound to YWHAZ and upregulated 14-3-3ζ expression in HCC cells. The biological functions of 14-3-3ζ have been previously investigated, including regulation of glycolysis and drug resistance [28]. Moreover, high expression of 14-3-3ζ was associated with poor prognosis in HCC patients [29]. YWHAZ is an oncogene in HCC that enhances the malignant properties of cells [30]. For example, 14-3-3ζ enhanced sorafenib resistance [11]; activation of the PXN/YWHAZ/AKT pathway accelerated cell cycle progression in HCC [31]. In addition, 14-3-3ζ aggravated the malignant progression of tumors by regulating glycolysis [32, 33]. The current study suggested that KHDRBS3 regulates proliferation, apoptosis, and glycolysis in HCC by binding to YWHAZ and upregulating 14-3-3ζ expression.

## Conclusion

To conclude, our data provides evidence to support that KHDRBS3 facilitates HCC progression through upregulation of 14-3-3ζ, which is associated with cell proliferation, apoptosis, glycolysis, and chemoresistance. Our findings highlight a novel mechanism of KHDRBS3 in HCC progression and provide the KHDRBS3/14-3-3ζ axis as a promising drug target for the treatment of HCC.

### Electronic supplementary material

Below is the link to the electronic supplementary material.

Supplementary Material 1

## Data Availability

All data in this study are available from the corresponding author upon reasonable request.

## Abbreviations

HCC: Hepatocellular carcinoma
KHDRBS3: KH RNA binding containing domain, signal transduction associated 3
DEGs: Differentially expressed genes
FC: Fold change
GO: Gene Ontology
CC: Cellular component
BP: Biological process
MF: Molecular function
IHC: Immunohistochemical
qPCR: Quantitative real-time-PCR
RIP: RNA immunoprecipitation
RFS: Recurrence-free survival
PFS: Progression-free survival
DSS: Disease-specific survival
SD: Standard deviation
