# Supplementary material for: KHDRBS3 accelerates glycolysis and promotes malignancy of hepatocellular carcinoma via upregulating 14-3-3ζ
Source: Cancer Cell Int. 2023 Oct 17;23:244. doi: 10.1186/s12935-023-03085-4 (PMC10583372; doi:10.1186/s12935-023-03085-4)
Supplement: Supplementary file 1 — Supplementary Material 1 [file 12935_2023_3085_MOESM1_ESM.docx]

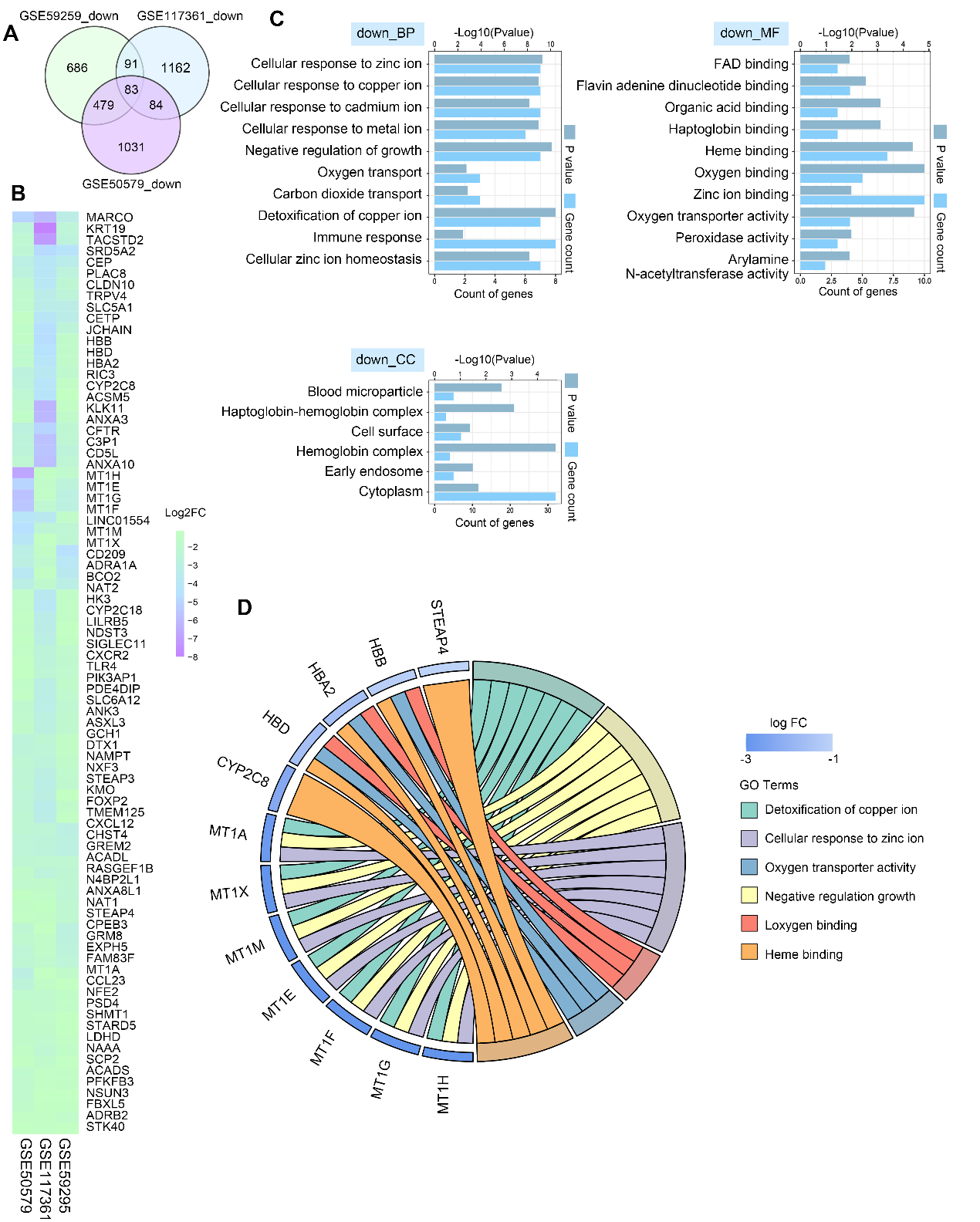


**Fig. S1** Identification and functional annotation of the downregulated differentially expressed genes in HCC.

(A) The Venn diagram suggests the overlapping downregulated DEGs among three datasets. (B) The heatmap diagram shows the overlapping downregulated DEGs. (C) GO analysis for the overlapping downregulated DEGs. The enrichment terms of biological process (BP), molecular function (MF), and cellular component (CC). (D) The chord plot shows the GO function enrichment. The left half ring shows the downregulated DEGs and the right half ring shows GO terms.
